# SUV normalisation and reference tissue selection for [^1^⁸F]mFBG PET-CT in paediatric and adult patients

**DOI:** 10.1007/s00259-025-07242-x

**Published:** 2025-04-02

**Authors:** Atia Samim, Diederik P. D. Suurd, Rob van Rooij, Max M. van Noesel, Marnix G. E. H. Lam, Arthur J. A. T. Braat, Nelleke Tolboom, Lise Borgwardt, Godelieve A. M. Tytgat, Bart de Keizer

**Affiliations:** 1 Princess Máxima Center for Pediatric Oncology, Utrecht, The Netherlands; 2 Department of Nuclear Medicine and Radiology, University Medical Center Utrecht, Utrecht, The Netherlands; 3 Department of Clinical Physiology and Nuclear Medicine, Copenhagen University Hospital-Rigshospitalet, Copenhagen, Denmark; 4 Department of Genetics, University Medical Center Utrecht, Utrecht, The Netherlands

**Keywords:** [^18^F]mFBG, Positron Emission Tomography, Standardised Uptake Value, Body weight, Lean body mass

## Abstract

**Purpose:**

*Meta*-[^1^⁸F]fluorobenzylguanidine ([^1^⁸F]mFBG) PET-CT is a novel imaging modality for norepinephrine transporter-expressing tumours, such as neuroblastoma and phaeochromocytoma, enabling quantitative assessment and improved diagnostic accuracy compared to *meta*-[^123^I]iodobenzylguanidine ([^123^I]mIBG) scintigraphy. This study aims to: 1) Identify the optimal standardised uptake value (SUV) normalisation method: body weight (BW) or lean body mass (LBM); 2) Determine the most stable reference tissue with SUV uptake below pathological levels.

**Methods:**

We analysed 63 [^1^⁸F]mFBG PET-CTs from 35 patients (20 paediatric neuroblastoma, 15 adult phaeochromocytoma). SUV_mean_ was measured in the liver, blood pool, bone marrow, and muscle, normalised using BW (SUVBW), LBM via James (SUVLBMJames), and LBM via Janmahasatian (SUVLBMJanma). Variability of SUVs and their correlation with weight were assessed.

**Results:**

LBM-based normalisation reduced SUV variability compared to BW-based normalisation. Bone marrow demonstrated the lowest variability and least weight dependency (r^2^ 0.45 for SUVBW versus 0.31 for SUVLBMJanma). The liver had the highest SUVs, increasing the risk of false negatives if used as reference tissue, while the blood pool had the lowest SUVs, raising the risk of false positives. Muscle showed relatively stable SUVs with increasing weight but higher variability than bone marrow.

**Conclusion:**

LBM-based SUV normalisation reduces weight dependency for [^1^⁸F]mFBG PET-CT. Bone marrow is the most reliable reference tissue due to its low variability and balanced SUVs, while muscle may serve as an alternative if diffuse bone marrow uptake is present. These findings support standardising LBM-adjusted SUV methods and using bone marrow as the primary reference tissue to enhance diagnostic accuracy.

Clinical trial registration: **EudraCT Number**: 2019–003713-33; **EU Clinical Trials Number**: 2024–513622-35–00.

## Introduction

*Meta*-[^1^⁸F]fluorobenzylguanidine ([^1^⁸F]mFBG) PET-CT is a novel diagnostic imaging modality to detect norepinephrine transporter (NET)-expressing tumours, such as neuroblastoma and phaeochromocytoma [1]. [^1^⁸F]mFBG PET-CT represents a significant improvement over standard *meta*-[^123^I]iodobenzylguanidine ([^123^I]mIBG) scintigraphy/SPECT-CT, combining the biological specificity of NET imaging with the superior spatial resolution and quantitative capabilities of PET imaging. Unlike gamma camera imaging used for [^123^I]mIBG, PET provides high-resolution and full-body 3D imaging. Studies have demonstrated the high sensitivity of [^1^⁸F]mFBG PET-CT for detecting pathological uptake in neuroblastoma and phaeochromocytoma [2–5].

Current [^123^I]mIBG imaging relies heavily on visual evaluation using established scoring systems such as the SIOPEN (International Society of Paediatric Oncology Europe Neuroblastoma) and Curie [6–8]. These systems, developed for planar [^123^I]mIBG scintigraphy, depend on visual interpretation, comparing pathologic tissue to background tissue; and are limited by [123I]mIBG's low resolution and lack of quantitative precision. [^1^⁸F]mFBG PET-CT overcomes these limitations, with its superior resolution, higher signal to background noise ratio, and ability to accurately quantify uptake. In the study by Samim et al. [2], the SUV_max_ for neuroblastoma lesions was 4.7 ± 3.5 (Interquartile range: R 2.1–7.7), with values being normalized for lean body mass (LBM). Meanwhile, in the study by Wang et al. [3], tumour-to-background ratios (TBRs) were used instead of direct standardised uptake values (SUVs), where muscle was used as the background reference tissue.

Quantitative comparisons of suspicious uptake with standardised reference tissues add confidence and consistency to visual interpretation, improving diagnostic accuracy and response evaluation. For example, in [^1^⁸F]FDG PET-CT, response criteria, such as the Deauville score, combine visual and quantitative assessments of lesion uptake relative to reference tissues such as the liver and blood pool [9]. A similar approach with [^1^⁸F]mFBG PET-CT could enhance the reliability and reproducibility in both clinical and research applications, underscoring the importance of understanding normal uptake in key reference tissues for accurate interpretation.

Quantitative PET imaging typically uses SUVs, which measure the ratio of radiotracer activity concentration in a volume of interest (VOI) to the average activity in the body. SUVs in lesions are compared to those in reference tissues to define pathological uptake [10]. Traditionally, SUV is normalised to body weight (SUVBW), but the low uptake of [^1^⁸F]mFBG in fat [2] limits the accuracy of SUVBW, particularly in patients with variable body composition. These limitations complicate comparisons of SUV measurements within individuals over time and between patients.

To address this issue, LBM-based normalisation methods, such as the James (SUVLBMJames) [11] and Janmahasatian (SUVLBMJanma) [12] models, have been introduced, differing in their estimation of LBM based on height, weight, and sex. LBM normalisation methods aim to account for variations in body composition by reducing the influence of fat tissue. These methods have demonstrated reduced body weight dependency, making SUVLBM a preferred approach in [^1^⁸F]FDG PET imaging [13–18]. The same approach could offer similar advantages in [^1^⁸F]mFBG PET-CT imaging, particularly standardising measurements in diverse patient populations.

This study aims to enhance the reliability of [^1^⁸F]mFBG PET-CT quantification, supporting its broader application as a diagnostic and research tool in oncology. Standardising SUV measurements requires an understanding of normal variability in key reference tissues, such as the liver, blood pool, and bone marrow, which are frequently affected by neuroblastoma metastases. Specifically, this study aims to:

1. Determine whether SUVBW or SUVLBM provides the most consistent normalisation, minimising dependence on body weight.
2. Identify the most suitable reference tissue with the least variability and SUV consistently below pathological levels.

These findings will help refine [18F]mFBG PET-CT quantification, ensuring greater accuracy and consistency in clinical and research applications.

## Materials and methods

### Patients

This retrospective study included all patients from the University Medical Center Utrecht and Princess Máxima Center for Paediatric Oncology, Utrecht, the Netherlands, who underwent [^1^⁸F]mFBG PET-CT between July 2020 and August 2024. Multiple scans per patient were permitted. The scans were performed as part of clinical care or as part of the MFBG pilot study for neuroblastoma [2] / the MFBG phaeochromocytoma study (EUCT 2024–513622-35–00).

### Imaging

All PET-CT scans were performed on a Biograph Vision 600 PET-CT system (Siemens Healthineers). Whole-body imaging was performed 60 min after intravenous injection of 2.0 MBq/kg [^18^F]mFBG, with a minimum of 20 MBq. First, a low-dose CT scan was acquired with an automatic tube voltage selection and current modulation (reference: 100 kV, 20 mAs) using CARE kV and CARE Dose4D (Siemens Healthcare) for attenuation correction of PET data. Total body PET scanning was performed using continuous bed motion, scanning two passes at 1.6 mm/sec for head to pelvis and 3.2 mm/sec for lower limbs. PET images were reconstructed using point spread function and time-of-flight modelling, with four iterations, five subsets, and Gaussian filtering of 4.5 mm for adults and 4.0 mm for paediatric patients. Image reconstruction matrix was 440 × 440 resulting in 1.65 × 1.65 mm pixels. PET images were reconstructed to a slice thickness of 3 mm. Patient height was measured in the outpatient clinic prior to the scan, and body weight was recorded in the scanning room.

### SUV measurements

Measurements on the [^1^⁸F]mFBG PET-CTs were performed using the above-mentioned reconstructions and thin slice CT series. SyngoVia (Siemens, Erlangen, Germany) was used to perform the measurements. VOIs were manually drawn on liver [left and right lobe], blood pool [ascending aorta], bone marrow (4th lumbar vertebral body [L4]; or if affected the proximal tibia), and muscle (gluteus maximus). Patients with liver metastasis were excluded from liver measurements. SUV_mean_ was defined as the mean uptake of all voxels within the selected ROI. Measured SUVBW was used to calculate SUVLBMJames [11] and SUVLBMJanma [12].

### Statistical analysis

Medians and interquartile ranges (IQR) were used to summarize non-normally distributed data. Scatterplots were created to visualise the relationship between body weight and SUV measurements for the different SUV formulations. A simple linear regression model (y = a*x + b) was applied to these data. The regression slope (a) and intercept (b) coefficients were used, and the regression coefficient (a/b) to assess the relative SUV dependence on body weight, independent of the intercept b.

The coefficient of determination (r^2^) was calculated to assess the degree of correlation between SUV measurements and body weight in the linear regression model.

Statistical significance was set at p < 0.05, and all analyses were performed using IBM SPSS Statistics version 29. Graphs were plotted using GraphPad Prism version 10.1.0 for Windows, (GraphPad Software, Boston, Massachusetts USA, www.graphpad.com).

## Results

### Patient Characteristics

A total of 63 [^1^⁸F]mFBG PET-CTs were performed in 35 patients, comprising of 20 pediatric and 15 adult patients. All imaging procedures were successfully completed with no reported complications and no adverse events related to [^1^⁸F]mFBG administration in either pediatric or adult patients. The study cohort included 18 males and 17 females, with 17 scans performed in 15 pheochromocytoma patients and 46 scans in 20 neuroblastoma patients. A total of 13 patients underwent multiple scans: two pheochromocytoma patients underwent two scans each while 11 neuroblastoma patients underwent a total of 36 scans. Patient demographics showed a body weight range of 5.5 to 96 kg (median 19.3, IQR 16.9–57.2), height range of 59 to 188 cm (median 112, IQR 102–169), and BMI values from 13.3–32.6 (median 16.2 IQR 15.1–21.6) (Table 1).

**Table 1:** Patient Characteristics and Body Parameters

Characteristic	Total (*n* = 35 patients)	Total (63 scans)
Diagnosis (%)		
- Neuroblastoma	20 (57%)	46 (73%)
- Phaeochromocytoma	15 (43%)	17 (27%)
Sex (%)		
- Male	18 (51%)	
- Female	17 (48%)	
Body Parameters (Median (IQR))		
- Height (cm)		112 (102–169)
- Body weight (kg)		19.3 (16.9–57.2)
- Lean body mass, James (kg)		16.9 (14.3–47.7)
- Lean body mass, Janmahasatian (kg)		16.3 (12.8–44.4)
- BMI		16.2 (15.1–21.6)
Dose (Median (IQR))		
- Prescribed activity (MBq/kg)		2
- Administered activity (MBq)		40 (34–97.8)

### SUV normalisation: SUVBW vs SUVLBM

**Fig. 1:**
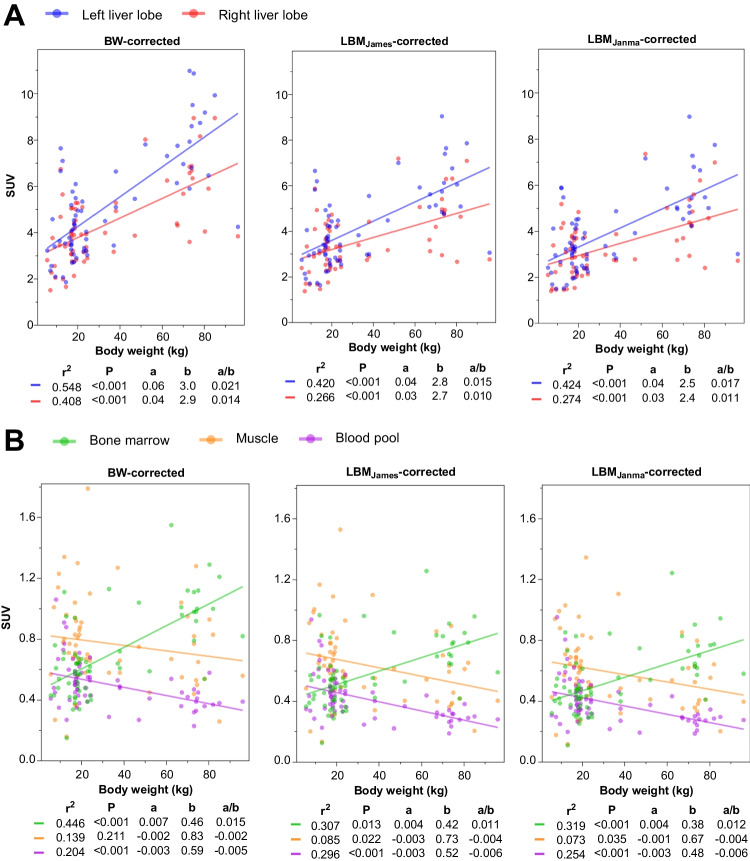
Scatterplots of mean Standardized Uptake Value (SUV_mean_) across background organs, corrected for Body Weight (BW) and Lean Body Mass (LBM). Data are presented for the liver lobes (**A**); and bone marrow, muscle and blood pool (**B**) corrected for BW, LBM according to James (LBMJames), and LBM according to Janmahasatian (LBMJanma). Linear regression lines for SUV measured in the left liver lobe and right liver lobe, with r^2^ representing the coefficient of determination and corresponding *P*-values of the Pearson correlation coefficient. The slope (a) and intercept (b) indicate the increase in SUV_mean_ per kg of body weight; a/b indicates the increase in SUV_mean_ per kg bodyweight irrespective of intercept

Correlations of SUV normalisation methods with body weight showed distinct patterns across tissues (Fig. 1). The left liver lobe, right liver lobe, and bone marrow all showed positive correlations with increasing body weight. In contrast, muscle and blood pool demonstrated weak negative correlations with body weight across all SUV normalisation methods. The left liver lobe exhibited the strongest positive correlation among tissues (r^2^ 0.55 for SUVBW). This strong positive correlation reduced when corrected for LBM (r^2^ 0.42 for SUVLBMJanma). Similar reductions were observed also for the right liver lobe (r^2^ 0.41 → 0.27) and bone marrow (r^2^ 0.45 → 0.31). When comparing the decrease in a/b ratio, the left liver lobe showed to largest reduction: the a/b ratio decreased from 2.1% (SUVBW) to 1.5–1.7% (SUVLBM), while the right liver lobe showed a reduction from 1.4% (SUVBW) to 1.0%–1.1% (SUVLBM) and bone marrow from 1.5% (SUVBW) to 1.1%–1.2% (SUVLBM). For the blood pool, r^2^ ranged from 0.20 (SUVBW) to 0.30 (SUVLBMJames), with corresponding a/b ratios of −0.5% to −0.6%. Muscle exhibited consistently low r^2^ values (< 0.14) and a/b ratios between −0.2% and −0.4%.

### SUV variability in reference tissue

**Fig. 2:**
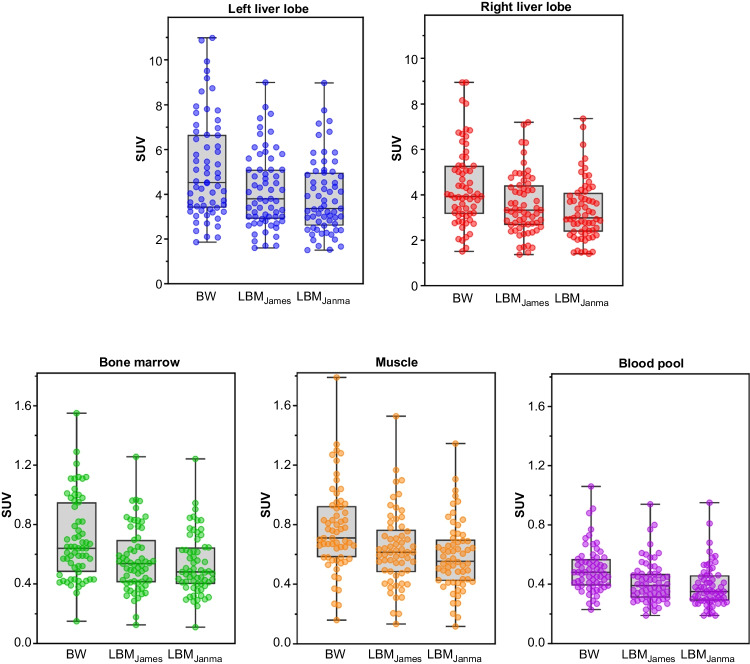
Mean Standardized Uptake Value (SUV_mean_) across background organs, corrected for Body Weight (BW) and Lean Body Mass (LBM), Boxplots display the range, median and interquartile interval for liver, bone marrow, blood pool, and muscle, corrected for BW, LBM according to James (LBMJames) and LBM according to Janmahasatian (LBMJanma)

The variability of SUV measurements in the liver, bone marrow, blood pool, and muscle, is summarised in Fig. 2 and Table 2. LBM-based normalisation demonstrated reduced variability compared to BW-based normalisation across all reference tissues.

**Table 2:** Mean Standardized Uptake Value (SUV_mean_) across background organs, corrected for Body Weight (SUVBW) and Lean Body Mass (SUVLBM) according to James (SUVLBMJames) and according to Janmahasatian (SUVLBMJanma)

Tissue	SUVBW	SUVLBMJames	SUVLBMJanma
Left Liver Lobe			
Median	4.53	3.80	3.37
IQR	3.40–6.66	2.90–5.10	2.60–4.97
Min–Max	1.86–11.0	1.60–9.00	1.50–8.98
Right Liver Lobe			
Median	3.93	3.33	2.98
IQR	3.16–5.28	2.67–4.42	2.37–4.09
Min–Max	1.51–8.95	1.37–7.19	1.41–7.35
Bone Marrow			
Median	0.64	0.54	0.48
IQR	0.48–0.95	0.41–0.70	0.40–0.65
Min–Max	0.15–1.55	0.13–1.26	0.11–1.24
Blood Pool			
Median	0.48	0.39	0.35
IQR	0.39–0.57	0.31–0.47	0.29–0.46
Min–Max	0.23–1.06	0.19–0.94	0.19–0.95
Muscle			
Median	0.71	0.61	0.55
IQR	0.58–0.93	0.48–0.77	0.42–0.70
Min–Max	0.16–1.79	0.13–1.53	0.12–1.34

Abbreviations

Blood pool had the lowest variability in SUVs, followed by bone marrow, which had a small IQR (0.399–0.646 for SUVLBMJanma). Liver and muscle exhibited larger IQRs compared to blood pool and bone marrow.

## Discussion

This study identified two key findings: (1) LBM-based SUV normalisation significantly reduces weight dependency compared to BW-based normalisation, and (2) bone marrow demonstrated low variability and SUVs below previously reported SUVs of tumour lesions and is therefore the most reliable reference tissue for visual analysis and comparison in [^1^⁸F]mFBG PET-CT imaging. If uptake exceeds normal bone marrow uptake, it may be considered pathological. These findings provide critical insights into optimising both quantitative and visual PET imaging in both paediatric and adult patients by addressing weight dependency and variability in SUV measurements.

SUVBW normalisation exhibited the highest variability and strongest correlation with body weight across all reference tissues, except the blood pool. In contrast, LBM-based normalisation significantly reduced weight dependency, providing improved consistency and precision. Both SUVLBMJames and SUVLBMJanma formulations yielded comparable results, with SUVLBMJanma showing slightly lower a/b ratios, indicating marginally greater independence from body weight. These differences, though statistically small, suggest that SUVLBMJanm may be preferable for achieving more consistent SUV measurements, particularly in diverse patient populations with varying body compositions.

These findings confirm that LBM-based normalisation is superior for quantitative analyses in [^1^⁸F]mFBG PET-CT imaging. Prior studies on [^1^⁸F]FDG PET imaging in adult populations have demonstrated similar advantages of LBM adjustments in reducing weight dependency [13–15, 18, 19]. Our findings expand knowledge to paediatric patients, where limited research exists on SUV normalisation methods, and align with recommendations from PERCIST 1.0 for oncologic PET imaging [10]. Direct LBM measurements from CT scans are not yet available in PET-CT analysis. For this study, we used SUVBW, SUVLBMJanma, and SUVLBMJames because they are routinely available in PET viewing software. Future advancements in automatic CT-based LBM estimation could refine SUV normalisation. Although predictive LBM models such as James and Janmahasatian improve SUV normalisation, direct PET-CT-derived LBM measurements could provide a more individualised and precise alternative. Future developments in automatic CT-based LBM estimation may allow for real-time assessment of body composition, particularly in patients undergoing treatment-related muscle loss or growth-related changes in pediatric populations.

Similar to [^1^⁸F]FDG, we did not observe any measurable uptake of [^1^⁸F]mFBG in adipose tissue. This absence of uptake explains why SUVBW tends to overestimate SUV values, particularly in patients with higher fat mass, as the BW-based formula includes non-metabolic adipose tissue, leading to artificially inflated SUV measurements. In contrast, LBM-based normalisation provides a stronger correlation with reference tissues because it accounts only for metabolically active compartments of the body, thereby reducing variability and weight dependency. This further reinforces the use of LBM-adjusted SUV methods for more accurate quantification in [^1^⁸F]mFBG PET-CT, particularly in patients with variable body compositions.Although both LBM normalisation methods (SUVLBMJanma and SUVLBMJames) reduced variability and weight dependency compared to BW normalisation, only minor differences were observed between these LBM normalisation formulas. For instance, SUVLBMJanma consistently resulted in slightly lower a/b ratios compared to SUVLBMJames across all reference tissues, suggesting marginally greater weight independence. However, these differences were minor.

The differences in SUV uptake across reference tissues have important implications for [^1^⁸F]mFBG PET-CT quantification. The liver exhibited high uptake, often exceeding lesional SUV levels, making it unsuitable as reference tissue due to the risk of overestimating pathological uptake. Additionally, unexplained differences in uptake between the left and right liver lobes, along with metabolic activity variations and physiological fluctuations, may introduce additional inconsistencies. The blood pool had the lowest SUVs, potentially leading to false positive bone marrow assessments if used as a reference tissue. In contrast, bone marrow exhibited stable and reproducible SUVs, with minimal dependency on body weight, supporting its use as the preferred reference tissue in cases where diffuse bone marrow involvement is not suspected. However, if diffuse or multifocal bone marrow involvement is present or suspected, as is often the case in neuroblastoma patients, muscle serves as a good alternative reference tissue. Muscle displayed moderate uptake levels with relative low variability. These findings highlight the importance of selecting a reference tissue that minimises interpatient variability while maintaining reliable SUV quantification.

The integration of LBM-adjusted SUVs and bone marrow as a reference tissue for [^18^F]mFBG PET-CT has the potential to enhance therapy response assessments in neuroblastoma and phaeochromocytoma. LBM-adjusted SUVs reduce variability and body weight dependency, offering more consistent quantitative measurements within patients over time and across patients. Bone marrow, with its low variability and balanced uptake, provides a robust baseline for detecting changes in pathological uptake, particularly in skeletal metastases. Standardising LBM-adjusted SUV measurements and using bone marrow as a reference tissue may improve response assessment by reducing variability in SUV quantification. This could enhance the accuracy of distinguishing physiological uptake from residual disease and support the development of standardised response criteria in multicentre trials. These advantages may facilitate improved accuracy in treatment monitoring and outcomes assessment in both paediatric and adult oncology patients.

This study benefits from a relatively large cohort of [^1^⁸F]mFBG PET-CT imaging, including both paediatric and adult patients with diverse demographics in terms of age, gender, and tumour types. Standardised imaging protocols ensured consistency across datasets, enhancing the reliability of the findings. However, several limitations must be noted. Including two different tumour types may have influenced results, however, the sample size was insufficient for stratification by tumour type. Additionally, the overall sample size (63 scans from 35 patients), particularly the paediatric subgroup (46 scans from 20 patients), may limit the statistical power of the findings. While this study provides valuable initial insights into SUV normalisation and reference tissue selection, a larger multicenter dataset would strengthen statistical robustness and generalisability. Future studies should aim to validate these findings in larger cohorts, such as those reported in Wang et al. for neuroblastoma patients, to further support clinical applicability [3]. Multiple scans per patient may have introduced bias, although these reflect distinct functional or metabolic states during treatment. Undetected lesions in reference tissues, despite exclusion of patients with liver metastases, could have influenced SUV measurements. Blood pool measurements may have been less reliable due to smaller sampling volumes. While the James and Janmahasatian formulas are widely used for SUV normalisation, they were developed based on adult body composition data and do not fully account for the dynamic changes in muscle and fat proportions during pediatric growth. The Peters formula has been proposed as a more suitable alternative for children under 14 years old, as it is based on extracellular fluid volume estimation rather than weight-based models [20]. However, normalisation to the Peters formula is not currently available in standard PET viewing software, which limits its practical implementation in clinical imaging. Future research should explore the integration of pediatric-specific LBM models, such as the Peters formula, into PET software to improve SUV normalisation accuracy in young patients. While the Peters formula provides a pediatric-specific alternative to adult-derived LBM models, direct CT-based LBM measurements would allow for a more precise, individualized approach in both pediatric and adult patients. The feasibility of integrating both paediatric and imaging based assessments into PET analysis workflows should be explored.

Additionally, our study did not explicitly assess gender-based differences in SUV normalisation, although the James formula applies different mathematical models for males and females. Variations in weight coefficients and quadratic adjustments could introduce minor differences in SUV calculations, particularly in pediatric patients undergoing developmental changes. While previous studies suggest that James-based LBM estimations may perform slightly better in females[18], further research is needed to determine whether gender-specific differences in LBM estimation significantly affect SUV normalisation in [^1^⁸F]mFBG PET-CT. Future studies with larger cohorts should explore this aspect to refine SUV normalisation strategies further. Furthermore, the assumption that norepinephrine transporter (NET) expression remains constant may not account for potential physiological changes in [^1^⁸F]mFBG uptake with age or weight. Finally, differences in reconstruction protocols between adults and paediatric patients (4.0 mm vs. 4.5 mm Gaussian filtering) may influence SUV_max_ and SUV_peak_. Moreover, variations in PET-CT reconstruction algorithms across different imaging centers may further impact SUV comparability. The European Association of Nuclear Medicine (EANM) has developed updated harmonisation strategies through the EANM Research Ltd (EARL 2) program to improve cross-scanner reproducibility [21]. Future studies should validate SUV normalisation methods across multiple PET-CT systems in accordance with these standardisation guidelines to enhance consistency and clinical applicability. However, SUV_mean_, which was the primary focus of this study, is less sensitive to such differences, minimizing their impact on the study's conclusions. Future studies could further investigate the influence of gender-specific body composition differences and reconstruction protocols on SUV variability, particularly for SUV_max_ and SUV_peak_.

## Conclusion

SUVBW should be avoided for quantitative analysis and SUV measurements in [^1^⁸F]mFBG PET-CT imaging due to its strong dependency on body weight, leading to significant variability and overestimation in heavier patients. SUVLBMJanma is the preferred method due to its minimal weight dependency and reliability in both paediatric and adult patients. Bone marrow should be considered the standard reference tissue for [^1^⁸F]mFBG PET-CT imaging due to its low variability and independence from body weight. In cases of diffuse bone marrow involvement, muscle may serve as a secondary reference tissue. Implementing LBM-adjusted SUV normalisation, particularly SUVLBMJanma, could enhance quantitative PET assessment in both paediatric and adult oncology patients.

## Data Availability

The datasets generated and/or analysed during the current study are not publicly available due policy reasons but are available from the corresponding author on reasonable request.
